# Supplementary material for: Polymorphisms in Pfkelch13 domains before and after the introduction of artemisinin-based combination therapy in Southwest Nigeria
Source: PLoS One. 2025 Mar 31;20(3):e0316479. doi: 10.1371/journal.pone.0316479 (PMC11957316; doi:10.1371/journal.pone.0316479)

Samples: 12576
Bases: 1059
Average spacing: 12

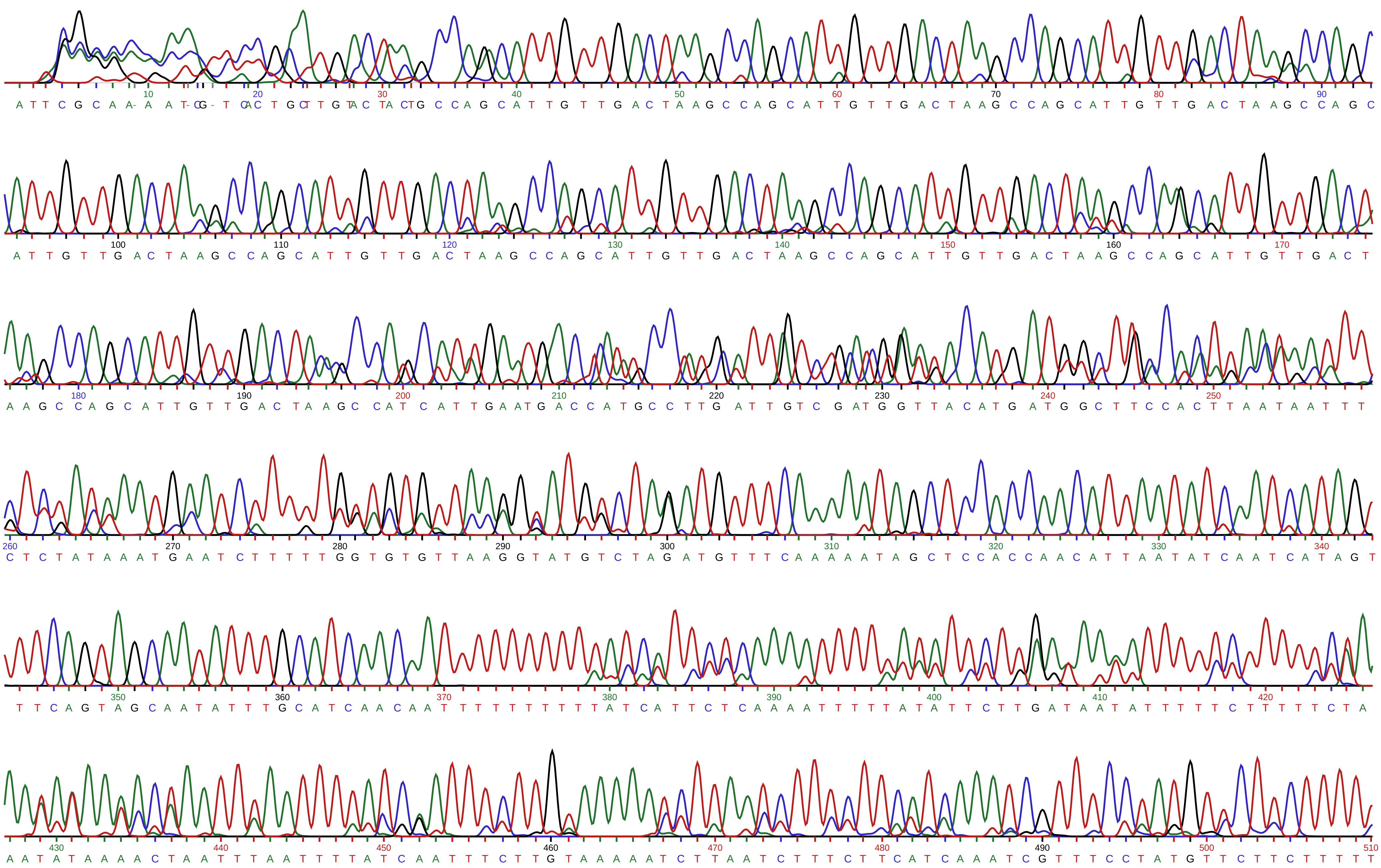

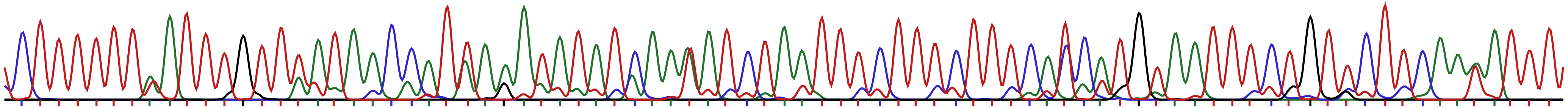

520 530 540 550 560 570 580 590

C T T T T T T A A T T T G T T T A T A A C C A T T A G A T A T A T C A A T A T C T A A T T T C T T T C T T T C A T C A T G T A A T T T C T G T T C T T C A A T A T T T

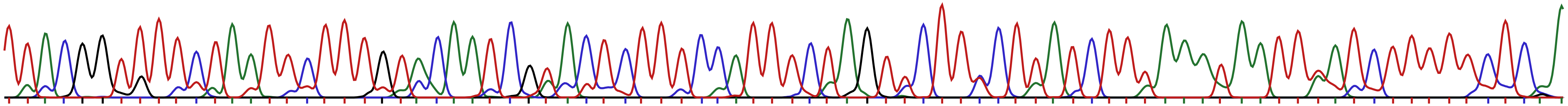

600 610 620 630 640 650 660 670

T T A C G G T T T T C T A A T T C T T T G T A C A A T C G T A C T C T T T C C A T T T C T A G T T C T T C C T T A T C T T T A A A T A A T T T A T C T T T T T C T C A A

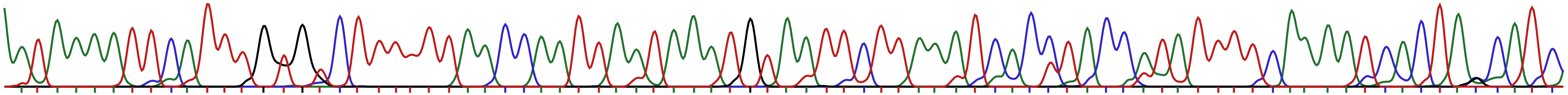

680 690 700 710 720 730 740 750 760

A T A A A A T T C A T T T G T G T C T T T T T T A A C C A A T T A A T A A A T G T A A T T C T T A A A T C A C C T A C C A T A T T T T C A A A A T C A C T A C C A T C

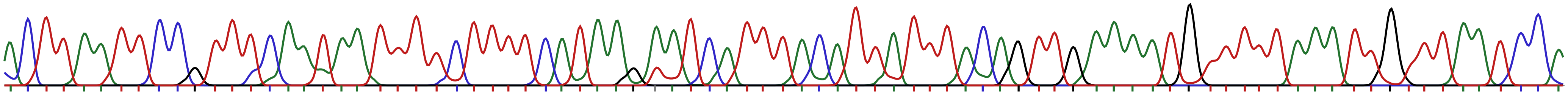

770 780 790 800 810 820 830 840

A C T T A A T T C C G T T T C A A T A A T T T T C T T T T C A T A A G - A T C A T T T A C A T T A T T T A C A G T T G A A A A T G T T T T T A A A T T G T T T A A T C C A

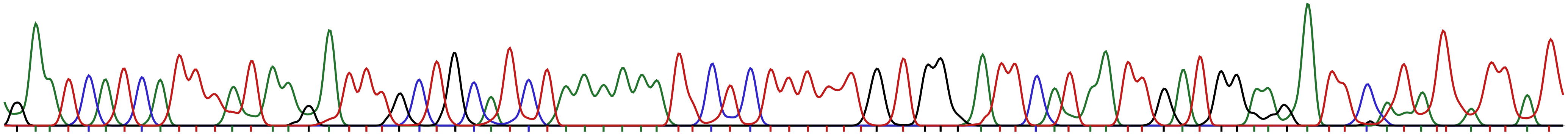

850 860 870 880 890 900 910 920

G A A T C A T C A T T T A T A A G A T T T G C T G C A T C T A A A A A A T T C T C T T T T T T G T G G G A T T C A T A A T T G A T G G A A G A T T C A T A T T A T T A T

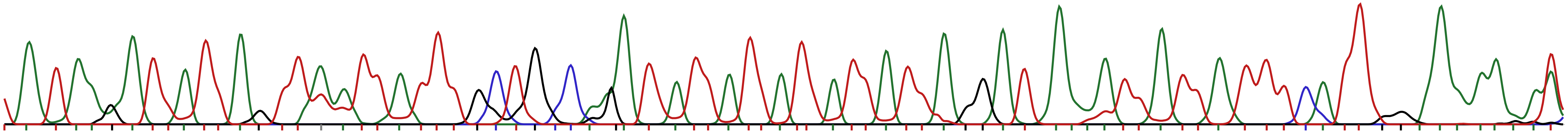

930 940 950 960 970 980 990 1000 1010

T A T A A G A T T A T T A G T T - A T T A T T T G C T G C C A G A T A T T A T T A T T A T T A T T A G G A T A A A A T T A T T A T T T C A T T G G A A A A A A A T

Samples: 12576
Bases: 1059
Average spacing: 12

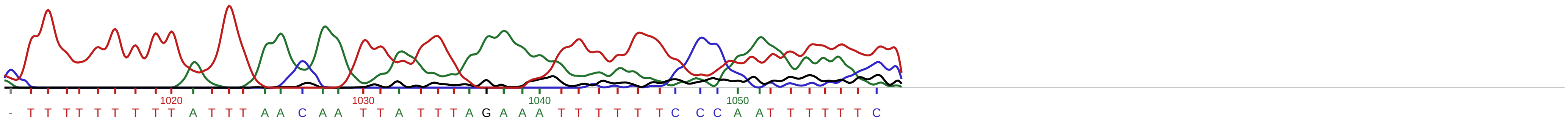

Supplement: Supporting information 2 — (ZIP) [file pone.0316479.s002.zip › 141KN2R_PREMIX_Plate_KELCH2_H02.pdf]
